# Pedestrian street behavior mapping using unmanned aerial vehicles. A case study in Santiago de Chile

**DOI:** 10.1371/journal.pone.0282024

**Published:** 2023-03-29

**Authors:** Daniel Parra-Ovalle, Carme Miralles-Guasch, Oriol Marquet

**Affiliations:** 1 Geography Department, Autonomous University of Barcelona, Barcelona, Spain; 2 Institute for Environmental Science and Technology, Autonomous University of Barcelona, Barcelona, Spain; University of Sheffield, UNITED KINGDOM

## Abstract

Objective observation of pedestrian behavior on the street has traditionally been difficult due to intensive commitment of time and resources with spatial analysis of pedestrian locations encountering additional problems. Recently, Unmanned Aerial Vehicles (UAVs) have gained popularity due to the significant improvements they offer over other conventional observation systems, such as their ability to cover larger surface areas in less time. This study tests the performance of UAV-based observation techniques in measuring pedestrian activity in two comparative settings in Santiago de Chile. The study develops an alternative technique adapting the behavioral mapping methodology that allows acquiring information about the people’s activities and the places where they are carried out. In this study a set of streets in the city of Santiago de Chile was selected as a case study, and the reliability of those observations was tested among raters in a population sample. Further, the use of a Geographic Information System (GIS) in the data coding process is detailed and exemplified using some of its spatial analysis tools. The results show high levels of inter-rater reliability in the different categories of recorded data. Finally, we discuss the advantages and limitations in observing pedestrian behavior using this technology and observation technique.

## Introduction

The investigation of people’s behavior in the street is acquiring increasingly more relevance due to the consolidated evidence related to walking with improvements in health, sustainability, and the creation of safer environments. While there is walking for utilitarian purposes, there is also evidence of important benefits in other street activities related to leisure and free time, such as children’s play, dog walking, jogging, or simply resting. Hence, a growing body of literature studies the relationship between the urban environment and people’s behavior on streets and in public spaces [1–3]. In this sense, in addition to the urban characteristics at the larger scale, the importance of micro-scale factors of urban design in promoting activities on the street, such as the quality of sidewalks, trees, benches, or playgrounds for children is discussed. For these reasons, in urban planning and design, there is a need to collect data on the activities of people on the street and detailed information on the locations of those activities.

Direct and systematic observation is one of the classic and essential methods for studying activities in public spaces [4, 5]. One of these observation methods, behavior mapping, allows researchers to study the behavior of people focus their interest on people’s relationship with the built environment, thus allowing to evaluate in detail the activities carried out and the interaction with the physical characteristics of places [6, 7]. But this methodology, when used in large urban environments such as parks, squares, or streets, has encountered various limitations inherent in traditional observation methods, such as the intensive commitment of time and resources, the collection of imprecise data, or the involuntary errors and fatigue of the observer, together with the difficulty of recording data from large population groups [8, 9].

Recent advancements in technology have greatly enhanced the ability to collect data on pedestrian behavior, through the use of Global Positioning Systems (GPS), WiFi signal detection sensors of smart devices, and computer vision. These methodologies provide valuable insights into pedestrian counts, unique user routes, and navigation patterns. However, these technologies alone do not provide sufficient information on the specific activities and behaviors of individuals

Unmanned Aerial Vehicles (UAVs), also known as drones, are increasingly used in urban studies. The utilization of the contribution analysis methodology reveals that the use of UAVs is due to the significant improvements that this technology offers over other conventional systems, highlighting its lower costs, its high mobility, the convenience offered by the aerial view, and the ability to cover larger surface areas in shorter time [10, 11].

In this context, this article details an alternative technique for observing people’s behavior on the streets using the city of Santiago de Chile (in central Chile, western South America) as a case study. The study adapts the behavior mapping methodology and examines the use of a UAV in the collection of information with a focus on where people are located, the activities they carry out, along with some of the personal attributes of those people. In recent years, the use of UAVs has become popular in urban studies, where the greatest application is in the fields of transportation engineering and traffic engineering. However, to date, only a few studies have been able to explore the use of UAVs in observing people on the street.

## Literature review

### Behavior mapping on the street

The systematic study of human behavior in public spaces began in the 1960s and 1970s, with methodologies based on direct observation, where the error levels could be significant. Over time, the introduction of some techniques and tools have improved procedures and increased confidence in the acquired data. The use of portable devices, such as hand-held personal computers (also known as personal digital assistants, PDAs) or computer tablets, or photographic and video equipment provide the information with greater precision [7, 12]. Also, the equipment’s location in fixed format on rooftops and terraces [5] or in mobile format on foot or by vehicle [13], increased the reliability of the observations. On the other hand, Geographic Information Systems (GISs) have made it possible to encode a greater number of variables, record the location with greater precision, and facilitate the processing, analysis, and representation of the results [7].

The first methods of objective observation of public spaces began being developed by the fields of environmental psychology, sociology, and criminology, during the 1960s and 1970s. Within these fields, the first methods include behavior mapping, developed by Ittelson, Rivlin & Prohansky [14], and Systematic Social Observation (SSO), developed by Reiss [15]. They are methods that study the relationship of people and the built environment, but where behavior mapping records types and frequencies of general behaviors in association with particular sites, SSO is concerned with social and physical upheaval in neighborhoods. More recently, and from other fields, several methods have been presented that observe the behavior of people and their physical context, highlighting for its popularity the System for Observing Play and Recreation in Communities (SOPARC) [16]. This tool is focused on the level of physical activity of users, is frequently used in environments such as parks [17, 18], and is also implemented in street observation [19, 20].

Behavior mapping is a widely used method for direct and systematic observation of what people actually do and where they are distributed. It was initially applied in indoor environments, and later it has been frequently used in public spaces such as streets, parks, squares, and playgrounds [6, 7, 21]. During the 1970s and 1980s, this methodology was frequently used to study the behavior of children in their residential settings, with special interests in observing their recreational activities [22–24] and in the impact of traffic on these activities [25–27]. Currently, studies have been extended to other street designs and urban environments, in addition to new user groups and activities, such as the investigation by Mehta [2] of social activities in shopping streets.

Within this methodology, there are two important concepts: behavior setting and affordance. Developed by Barker (1968), the concept of behavior setting refers to spatial units where physical characteristics associated with a specific pattern of behavior have been identified [7, 8]. On the other hand, affordance, a concept introduced by Gibson [28], is defined as the “perceptible properties of the environment that have a functional meaning for an individual. […] They indicate what can be done in an environment and what activities can be ruled out.” [7]. In addition, Kytta [29] differentiates between passive affordances, referring to those that are perceived and can be revealed through self-report, and active affordances that are revealed through the actions of the individual as they are used and configured, and can be known through methodologies such as behavioral mapping.

Several authors indicate 5 necessary elements in the observation process: (1) a graphic representation of the observed areas; (2) a clear definition of the human behaviors observed, counted, described, or schematized; (3) a schedule of repeating times during which observation and recording take place; (4) a systematic observation procedure; (5) and a coding and counting system that minimizes the effort required in recording observations [8, 30–32].

Despite the positive assessment given to objective information, some authors point out that a decrease in observational studies has been observed in recent decades [8]. This paradox can be found in the limitations of traditional observation methods, such as the intensive commitment of time and resources, the collection of inaccurate data or data with involuntary errors and observer fatigue, together with the difficulty of recording data from large population groups [8, 9, 33]. In addition, it is noteworthy that people can modify their behavior if they become aware that they are being observed, due to how intrusive some of these methods can be [8].

### Unmanned aerial vehicles and observation of activities on the street

In recent years, the utilization of Global Positioning Systems (GPS) and WiFi signal detection sensors in mobile devices has provided a large amount of objective data on mobility patterns. Applications have been developed that leverage the precise location provided by the GPS of mobile devices to capture detailed information on specific routes and modes of transportation for subsets of pedestrians using the application [34, 35]. Similarly, WiFi signal detection sensors have been utilized to collect data on a broader subset of the population [36]. However, as different studies have highlighted, the population observed using these smart device-based methodologies is not necessarily representative of the general population. Other methodologies such as automatic pedestrian counts using sensors at specific points or computer vision techniques in video transmissions have attempted to address these limitations. Automatic counts based on various sensor technologies (infrared sensor, induction loop, radar sensor, etc.) have been used for several decades, providing long-term and detailed temporal data but are limited in their ability to cover larger areas and only provide information on the number of pedestrians. In recent years, techniques have been developed to detect people from video transmissions. Computer vision not only allows for counting and tracking the flow of people but also for detecting and following individual people, thereby providing information on routes and speeds of movement through public spaces [37]. Additionally, promising advancements have been made in recognizing people’s actions and activities [38].

UAVs are increasingly used in urban studies. Their main application is in the fields of transportation engineering and traffic engineering, consolidating the UAV as an alternative to traditional methods of collecting data related to motorized vehicles on streets and highways. However, recently, UAVs are starting to be used to examine new uses related to the study of pedestrian mobility and the behavior of people in the streets, such as the monitoring of pedestrian traffic in pedestrian-only shopping streets [39], pedestrian counts in urban streets [40], or the street audits to measure the fiscal disorder of a neighborhood [41].

The methodological contribution of the use of UAVs is due to significant improvements that this technology offers over other conventional systems, highlighting its lower costs, high movement, convenience offered by the aerial view of an area, and the ability to cover larger surface areas in shorter time [10, 11]. In addition, continuous improvements in computer vision in automating tasks, such as vehicle detection and tracking, have facilitated the processing and analysis of large amounts of data [42–44]. Pedestrian detection and tracking are even more difficult due to the relatively small size of people, the combined movement of pedestrians and the drone, and the occlusion generated by trees or other objects, which is why some studies have used semi-automatic techniques at the time of processing the pedestrian data of the videos [45–47]. Even more challenging are the tasks of automating the recognition of people’s activities and their individual characteristics [48].

Among the studies that explore UAV use in the acquisition of data for the study of human behavior on the street, Sutheerakul et al. [39] tested the capacity of the drone by applying it in a case study in a commercial street where they collected data from pedestrians. This method is based on traditional pedestrian information gathering techniques and subsequent manual data acquisition. Along with measuring the flow, speed, and density of pedestrians, they include the recording of some individual characteristics of people, such as gender, age group, or group size. However, Park & Ewing [40] tested a new pedestrian observation method using the UAV, based on a system that measures the volume of pedestrians on walking routes that was developed by Ewing & Clemente [3]. In that study, in addition to using the UAV to acquire estimated data on gender, age group, and mode of transportation, recorded street furniture and equipment, such as bus stops, bicycle racks, and benches. Finally, Grubesic et al. [41] present a method that captures information on the ecological characteristics of neighborhoods. Their study was based on SSO techniques and replaced walking tours in the neighborhood with the collecting of images using the UAV thus allowing them to build a high-resolution orthomosaic of the study area. The subsequent audit of the physical disorder of the public spaces of the neighborhood would be carried out using GIS or remote sensing software packages.

However, there are no studies that observe street behaviors in detail using UAVs, and that in addition to considering variables that allow the study of people’s mobility, such as flows or mode of travel, include objective data on other activities performed on the street, such as those related to leisure or work. Although recently Park, Christensen and Lee [49] explored the use of behavior mapping techniques using the UAV to observe park users. The present study develops a protocol that redefines the 5 elements of behavior mapping and adapts the SOPARC observation technique to its observation and data acquisition process.

## Methodology

Using the city of Santiago de Chile as a case study, we developed an alternative approach of systematic behavioral observation of daily activities in the street, based on the use of an UAV. We adapted the data acquisition techniques of behavior mapping to be: (1) centered on the place, (2) based on on-site observations, detailing the necessary steps in the collection of the information in the place, in the subsequent acquisition of the data, and (3) coding the data in a Geographic Information System (GIS).

### Study area and UAV used

**Fig 1:**
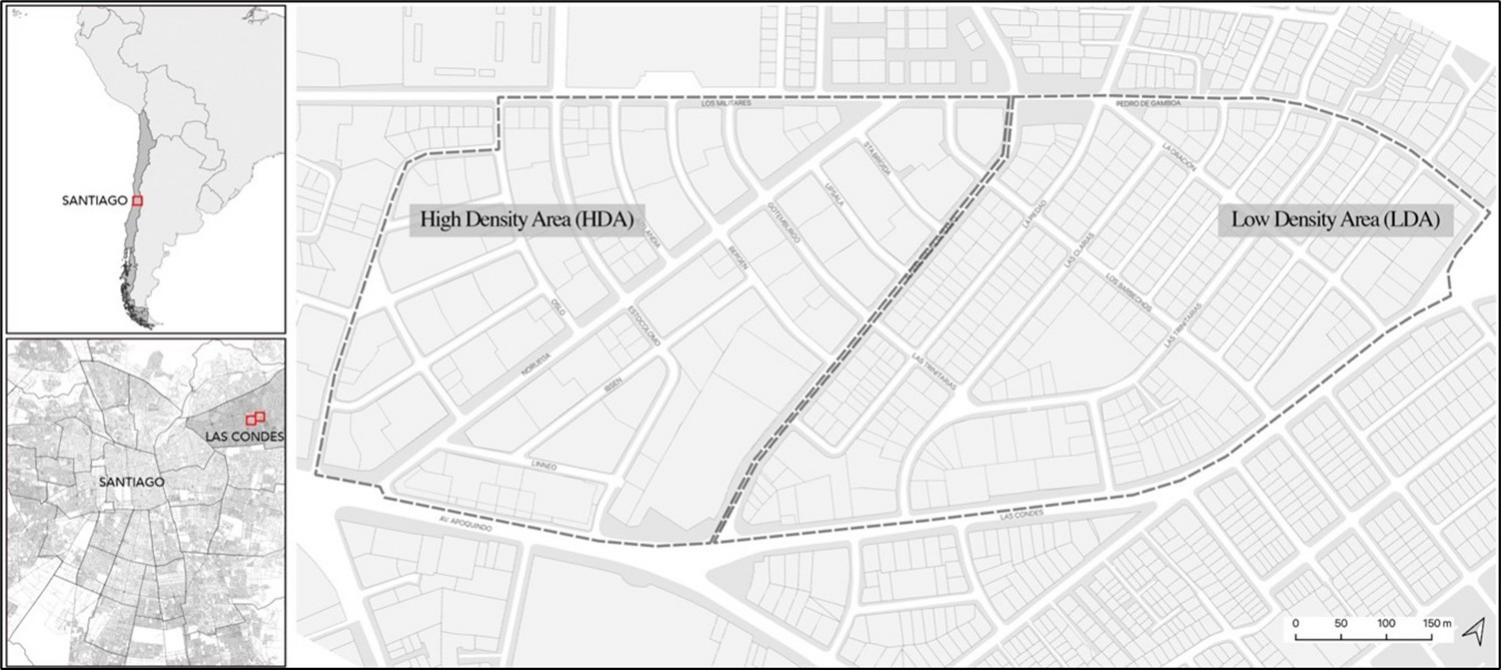
Observation areas. Image Source: own production based on Openstreetmap data.

For the pilot study, two contiguous areas were selected in Las Condes, a commune in the Northeast zone of the city of Santiago de Chile (Fig 1). The commune of Las Condes is an important metropolitan sub-center that is home to a great deal of financial and commercial activity as well as a large part of high-income social groups. Some areas of this commune have experienced an intense process of densification, where single-family homes have been replaced by high-rise residential buildings.

**Fig 2:**
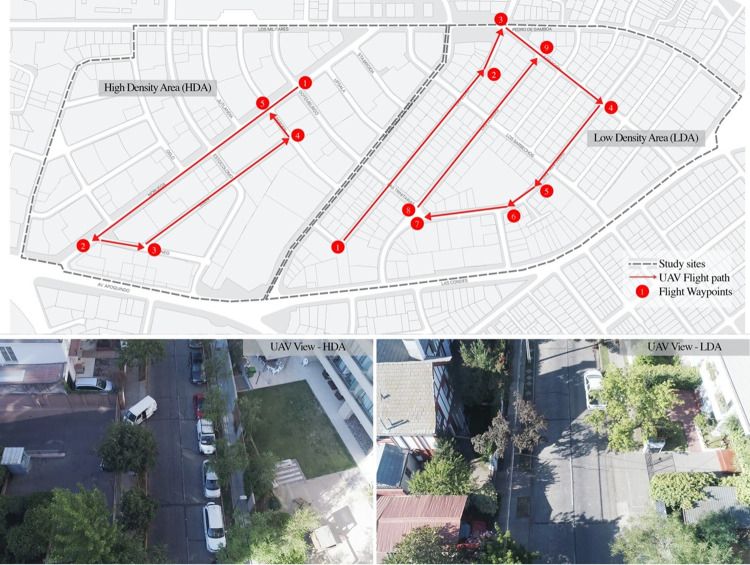
UAV flight path (top) and view from UAV (bottom). **Source**: own production based on Openstreetmap data and original UAV imagery.

For our observations we selected 4 sections of streets contiguous to each other in one of these densified neighborhoods (High Density Area, HDA), whose gross residential density in persons per hectare of land (pers./ha) reaches 275 pers./ha (INE, 2017) and 4 sections of streets in a tightly-packed area that largely maintains its original single-family homes, with a density of 62 pers./ha (Low Density Area, LDA) (Fig 2). The route through the four streets of the HDA is 1.1 km (0.68 mi) in total length and in the LDA the route has a total of 1.5 km (0.93 mi). Both sectors share some urban and social characteristics, such as the predominantly residential land use, the size of the blocks, the width of their streets, the accessibility to transport, and the socioeconomic level of their residents. The streets have a profile of 12 to 15 m (39.37 to 49.21 ft) in width, and are configured by a central two-lane expressway flanked by rows of trees on a strip of grass that separates the road from the paved sidewalks.

### Process of observing activities in the street using the UAV

For the study of street activities, a Phantom 4 Advanced UAV (DJI, Shenzhen, China) was used, with a built-in camera that allows video recording at a maximum resolution of 4096 x 2160 pixels and with a viewing angle of 84°. The camera was connected with a 3-axis stabilizer, which allows a tilt angle range from -90° to + 30°. The optimal flight autonomy specified by the manufacturer is 25 min, but in our previous tests we have determined an optimal duration of 15 to 20 min for data collection; 6 batteries were used.

Before operating the equipment, it was necessary to comply with the requirements of the local legislation that regulates the use of UAVs. In Chile, the General Directorate of Civil Aeronautics (DGAC) is the body that regulates air activity, including drone flights. Many of the requirements and restrictions on flights over urban areas in Chile are similar to other OECD (Organisation for Economic Co-operation and Development) countries [50], requiring UAV registration and certification, the operator having the proper credentials, contracting of insurance for damages against third parties, and the management of a permit to operate in populated areas. The present study met all the necessary requirements.

Five elements have been identified that are used in the observations process for the traditional behavior mapping methodology [8, 30, 31]. The adaptation of these five elements when using the UAV in observing behavior on the streets is detailed below.

1. *Define the types and categories of behavior*: As with the traditional method, it is necessary to define the types and categories of behavior along with monitoring other data relevant to the research problem. Three groups of data were defined that provide different types of information on behavior: Group 1 has estimated information on the characteristics of the subjects such as gender (male or female) and age group (child, adult, older adult); Group 2 is based on information that describes people’s behavior, such as postures (e.g., standing or walking) and activities (e.g., playing, traveling on foot, or walking the dog); Group 3 corresponds to the interaction of people with other people and with objects, for which we acquired information on the size of the group, the interaction with personal objects, such as baby carriages or dogs, and the interaction with means of transportation, such as bicycles.
2. *Schedule of times and their repetitions during which the observation takes place*: Our objective was to test the methodology by recording the moments with the greatest presence of people and also where activities of a different nature are carried out. After touring the observation area with the UAV at different times of the day, we defined 3 different hours for business days: from 13:00 to 14:00; from 16:00 to 17:00; and from 18:00 to 19:00. In each of these schedules, tours were made at the beginning of each of the three 1 hr periods and then a second flight was made 30 min later, adding up to a total of 6 flights for each of the routes.
3. *Systematic observation procedure*: The possibility of making automated observations when using the UAV guarantees a procedure with a greater degree of systematization, becoming one of its great advantages. The GO 4 app (DJI, Shenzhen, China) *for* drone pilots was used, that requires manually flying over the study area and thus marking the necessary waypoints on the different locations delimiting the flight routes. In addition, parameters of height, flight speed, and the angle of inclination of the camera were defined that would be repeated in each of the observations. Low-altitude flights allowed for greater detail in the videos, thus improving the perception of gender or age group of people. A minimum height of 25 m was established, which allowed to overcome the foliage of some trees present along the routes and record the total width of the streets. In addition, this height allowed discreet flights to be carried out without altering people’s behavior. The flight speed was 6 km/h (3.773 mph), similar to the walking speed for intermediate-level walkers, which allows the operator to maintain permanent visual contact with the UAV. The tilt of the camera was set at -60°, balancing the need to observe people in perspective, thus distinguishing their faces, together with a vertical observation that helps to discover people hidden by objects or tree foliage. The UAV flew 6 times on each of the two routes as planned, taking 12 min to complete each flight in the HDA and just over 16 min in the LDA. The flights were made between Tuesday, December 12, and Thursday, December 14, 2019.
4. *Base map that identifies the essential physical characteristics*: Before collecting the data, it was necessary to prepare a map of the study area in which the observed people were georeferenced. The videos obtained with the UAV are useful to validate or complete the maps with the essential physical characteristics of each study, such as, e.g., information on street crossings, urban trees, or bus stops. In addition, the synchrony between the data on people’s activities and the data on the physical characteristics of the place can be key to understanding the correlation between environment and behavior. For this reason, it was convenient to add information on temporary elements that alter activity on the street, such as closed sidewalks preventing pedestrian crossing due to public works, stopped vehicles, or obstacles such as fences or scaffolding. The base map of our observation area was prepared using the QGIS v3.10 software (i.e., Free and Open Source Geographic Information System).
5. *Coding and counting system*: The video data collection process resulted in 36 video files with 160 min duration in total. After ruling out non-useful parts such as the UAV takeoff and landing or turns between the street the video time dropped to 115 min. The information encoding process was carried out by manually reviewing these videos. A first operator used the GIS to georeference data points on the base map of all the people identified in the observed streets, including some who were hidden by tree foliage. For some of these cases, we amplified and improved the contrast of the images using video editing software (iMovie v10.1; Apple Inc., Cupertino, California, USA), which helped to identify the people. In addition, in order to facilitate future revisions, in this first instance the attributes of each point included the name and time-point of the video where the person appears, the degree of occlusion, and the direction in which they were traveling. Next, two evaluators jointly reviewed some of the videos, defining a list with the options for each variable, and unifying some of the criteria. These two raters then separately coded the attributes of gender, age group, group size, posture, activity, and interaction with personal items and transportation vehicles.

### Analysis and presentation of the results

Accuracy in recording observed activities may have some degrees of error, even when the data come from video sequences that allow for multiple revisions. Since inter-observer reliability is an important element of systematic field observation tools, one of the objectives of this research is to test the reliability of using a UAV to map street behavior, i.e., whether the tool can be used reliably to collect data on street activities. In this study we performed a reliability test between raters, using Cohen’s Kappa coefficient (Cohen, [51] which is frequently used in the literature in the evaluation of observation methods [52]. Cohen’s Kappa coefficient indicates the agreement between the data obtained among different evaluators, where a value of 1 indicates “perfect agreement”, values greater than 0.8 indicate “almost perfect”, and values between 0.6 and 0.8 indicate “substantial agreement”. Two evaluators (the principal investigator of this work and a second evaluator not involved in the project) looked at the UAV videos acquired in the HDA area and independently coded the different attributes and personal behaviors. Both evaluators are members of the Mobility, Transport and Territory Group (GEMOTT) of the Autonomous University of Barcelona. The analyses were carried out using the software R v1.3 (R Project for Statistical Computing).

The results that we present are only intended to be representative of the possibilities of analysis of this data type, and in no case are they intended to be exhaustive. Recognizing attributes and activities performed by people from video sequences is a difficult task due to problems such as partial occlusion, scale changes, point of view, lighting and appearance. These problems affect the recognition of variables in each category to varying degrees. For example, the video sequence in which a person’s face is hidden may make it impossible to recognize his or her sex, but not his or her posture. Consequently, the final data show different percentages of unidentified variables.

**Fig 3:**
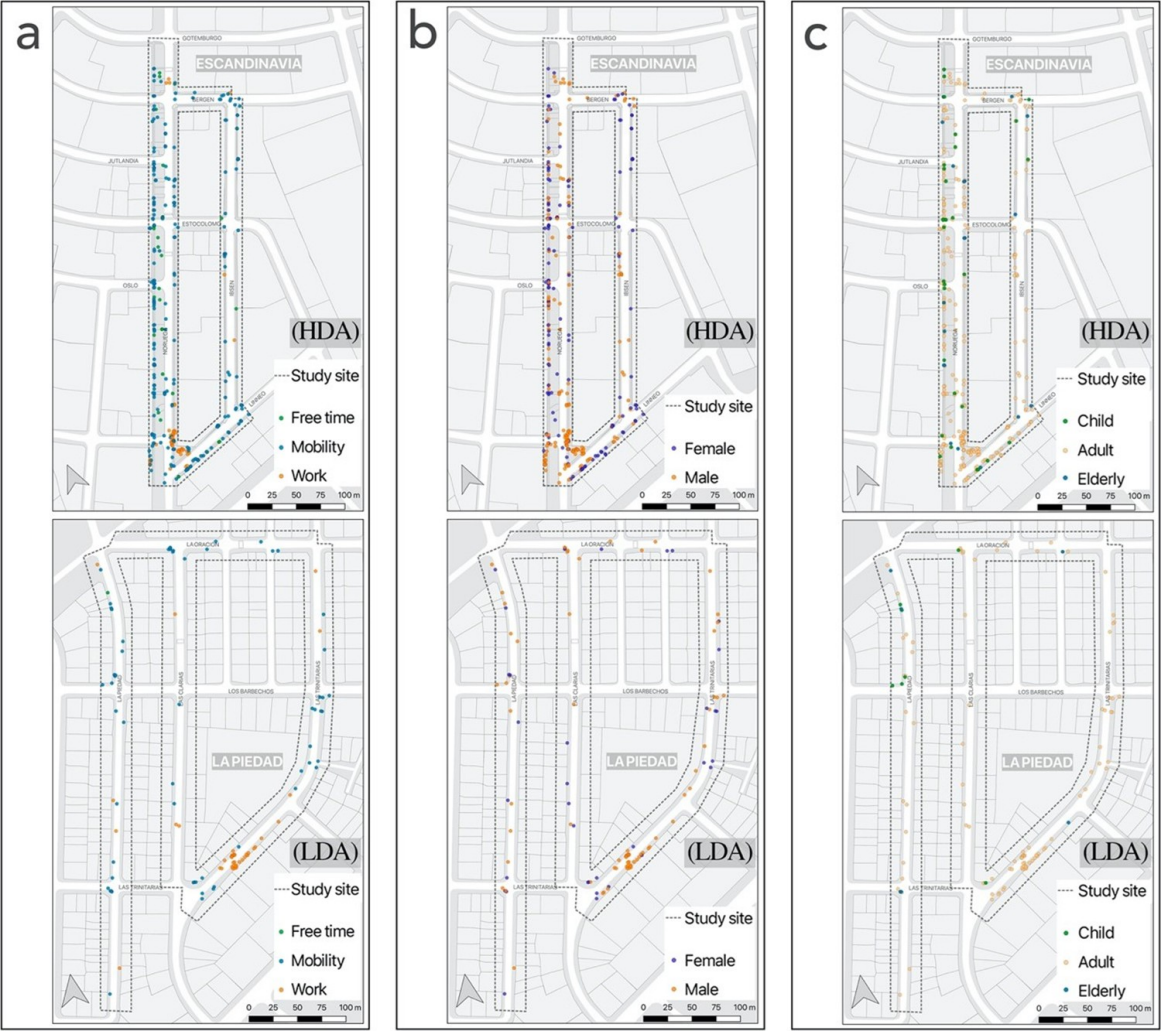
Spatial distribution according to activities (a), gender (b), and age group (c). **Source**: own production over Openstreetmap data.

**Fig 4:**
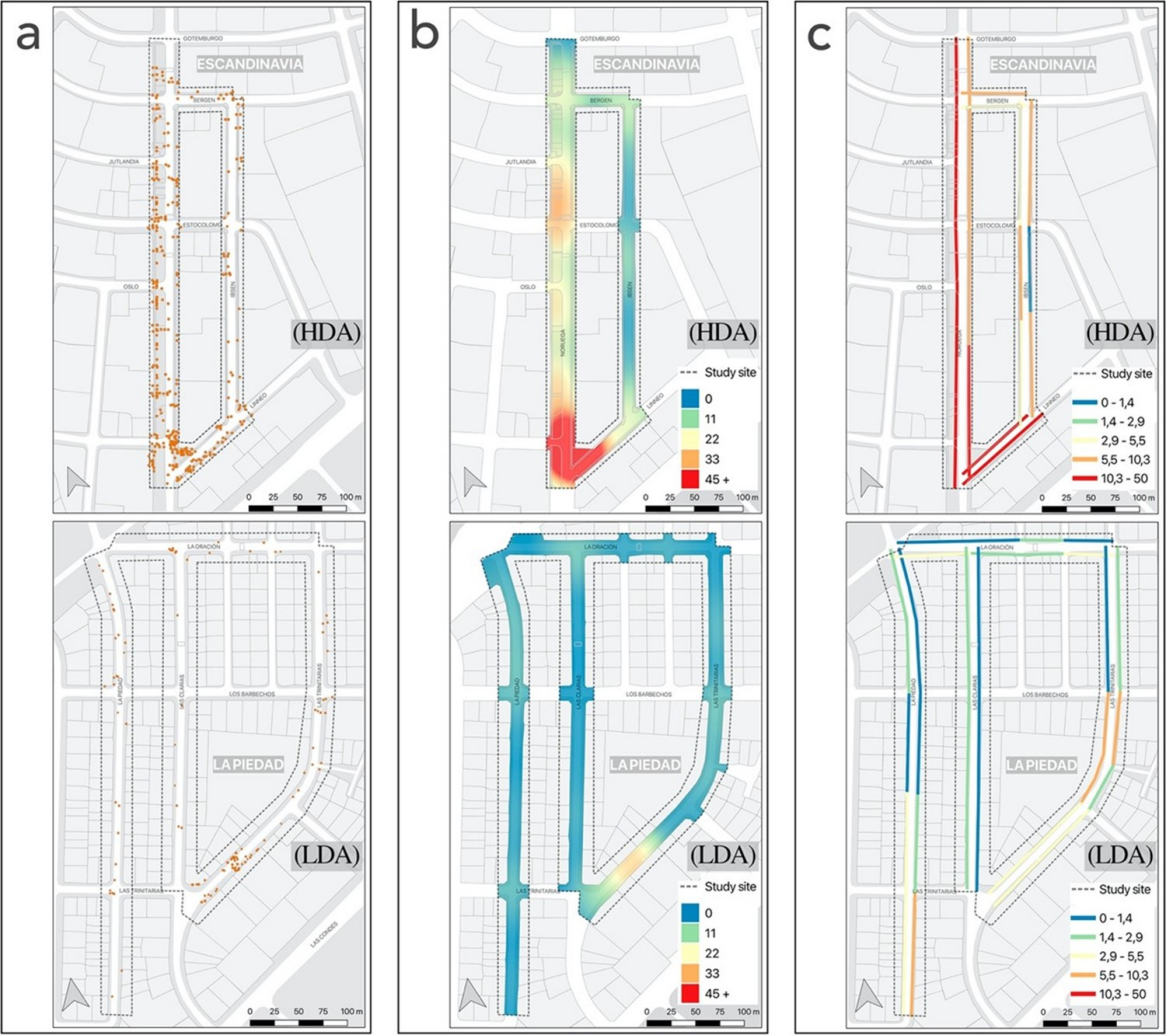
Point distribution (a), Kernel density (b) street segment (c). **Source**: own production over Openstreetmap data.

The reliability test results presented in Table 1 correspond to a subset of data observed by both evaluators and corresponding to the HDA area. A summary of the results based on the observed activities is presented in Table 2. Then, using GIS, some spatial analyses, that are frequently used in the literature were carried out on such as (1) the point distribution maps that illustrate the patterns of the use of space and (2) the Kernel Density Estimation map, for which we used the Heatmap tool in QGIS, and that allowed us to visualize the activity density in the street (via the density or heatmap raster of an input point vector layer). Both Table 2 and Figs 3 and 4 show the total results in the two observed areas (HDA and LDA) acquired by the principal investigator. Finally, together with a map and a table, the active affordances in one of the areas (HDA) are indicated, in relation to the typical behavior settings of a street, identified as the spatial units where their physical characteristics can be associated with general patterns of behavior (sidewalks, street intersections, bicycle lanes, roadways, and other public spaces).

**Table 1:** Results for Cohen’s Kappa agreement between two raters watching UAV video in HDA.

Category	average	% agreement	(κ) Kappa
GENDER	308	96.2	0.92
Male	174	97.0	
Female	134	94.2	
AGE GROUP	325	93.1	0.70
Child	29.0	81.3	
Adult	282.5	95.5	
Elderly	13.5	68.8	
GROUP SIZE	330.5	95.9	0.94
1	185.5	95.3	
2	102	100	
3	40.5	92.9	
≥ 4	2.5	25.0	
POSTURE	341	94.3	0.88
Walking	232	98.3	
Standing	61.0	93.7	
Lying	14.0	75.0	
Biking	13.0	85.7	
Sitting	11.0	69.2	
Riding	6.0	71.4	
Other*	4.0	75.0	
ACTIVITIES	306	93.5	0.93
Travel walking	207,5	95.8	
Building construction	23.0	91.7	
Resting	22.5	87.5	
Walking the dog	21.5	95.5	
Travel biking	13.0	85.7	
Travel scooter	6.0	71.4	
Delivery	4.5	80.0	
Household waste (collect)	3.0	100.0	
Other	5.0	80.0	
P. OBJECTS	34.5	93.3	0.85
Dog	21.5	95.5	
Baby stroller	7.0	100.0	
Shopping trolley	2.0	100.0	
Other	4.0	66.6	
T. VEHICLE	40.5	85.5	0.79
Bike	15.0	87,5	
Truck	9.5	90.0	
Scooter	8.0	100.0	
Car	6.0	71.4	
Motorcycle	2.0	33.3	

*Jogging, kneeling, holding object in arms

**Table 2:** Observed activities, gender, group age, and group size.

	Total		HDA	LDA	Gender		Group Age			Group Size		
Activities	All	%	All	All	Male	Female	Child	Adult	Senior	1	2	≥ 3
Mobility	298	65.6	238	60	125	146	36	228	23	181	87	30
Travel walking	263	88.3	214	49	104	142	24	205	23	160	78	25
Travel biking	21	7.0	14	7	16	3	3	18		17	2	2
Travel scooter	5	1.7	5		4		1	4		3	2	
Travel assisted	8	2.7	5	3	1		8				5	3
Travel wheelchair	1	0.3		1		1		1		1		
Work	91	20.0	50	41	88	3		89	1	30	22	39
Building construction	68	74.7	38	30	67	1		67		16	16	36
Cleaning (Manual sweep)	8	8.8		8	7	1		8		6	2	
Delivery	6	6.6	5	1	6			6		4	2	
Household waste (collect.)	3	3.3	3		3			3				3
Gardening	2	2.2		2	1	1		1	1	2		
Household waste (disposal)	1	1.1	1		1			1		1		
Other	3	3.3	3		3			3		1	2	
Free Time	34	7.5	31	3	15	16	2	29	1	27	7	
Walking the dog	23	67.6	22	1	8	15	1	21	1	22	1	
Resting	10	29.4	8	2	6	1	1	7		4	6	
Exercising (jogging)	1	2.9	1		1			1		1		
Unidentified*	31	6.8	24	7	15	8	1	24		15	13	3
Total	454	100	343	111	243	173	39	370	25	253	129	72
%	100		75.6	24.4	53.5	38.1	8.6	81.5	5.5	55.7	28.4	15.9

*e.g., standing doing something, occlusion

## Results

### Inter-rater reliability of UAV observations

Table 1 shows the results of the reliability analysis for each of the 7 groups of variables, according to Cohen’s Kappa coefficient. The agreement was “almost perfect” for group size with 0.94, for activities with 0.93, for gender with 0.92, for postures with 0.88, and for interaction with personal objects with 0.85. The concordance achieved “a substantial agreement” in the interaction with transportation vehicles with 0.79, and in age group with 0.70.

Traditionally, observers have difficulty grouping people by age based on their physical attributes. The boundaries between the different groups are often blurred and not objective. The low level of agreement in the case of older people (68.8) shows that this study does not escape this type of difficulty. On the other hand, the low level of inter-rater reliability in some specific categories, such as the category motorcycle (33.3) included in the group interaction with vehicles, and ≥ 4 (25.0) included in group size, may be due to the fact that these categories were recorded very few times, so that small disagreements end up strongly modifying the averages. In any case, the results of this study demonstrate that the UAV behavior mapping method is reliable.

### Observed activities

The number of persons observed in the HDA was 343, which together with the 111 persons observed in the LDA gave a total of 454 persons (Table 2). There were 14 activities identified that were classified into three categories: (1) Mobility Activities Category; this includes activities such as traveling on foot or traveling by bicycle which accounted for 65.6% of the total number of persons observed. (2) Work Activities Category; 20% of the persons observed carried out any of the 7 work activities, such as building construction, street cleaning (manual sweeping), distribution, household waste collection, gardening, and household waste disposal. (3) Free Time Activities Category; 7.5% of the persons were registered in one of the three activities in this category (walking the dog, resting, and exercising). Together with the predominance of mobility-related activities, which represent 68.2% in HDA and 54.1% in LDA, these results highlight the sharp contrast in the presence of work-related activities observed between the two areas, which represent 36.9% in LDA, compared to 14.6% in HDA. It was not possible to identify the activity in 6.8% of the cases. This was mainly due to the problem of partial occlusion, as well as the fact that some people were observed standing in an attitude of waiting for something, such as meeting someone, the arrival of a vehicle or simply wanting to stand in that place.

Regarding gender, a total of 243 men (53.5%) and 173 women (38.1%) were registered in the survey. The greater presence of men on the street is accentuated even more if we look at work activity, where men represent 96.7% of the total number of people who carry out work activities. In the Group Age Category, 8.6% of persons were children, 81.5% were adults, and 5.5% were older adults. Although the low presence of children may be partly explained by the fact that only one of the recordings took place outside school hours, adults observed on the street are overrepresented relative to the general population, indicating a problem in the city. In the Group Size Category, 55.7% of the persons carried out their activities individually, 28.4% were accompanied by a person, and 15.9% were registered in groups of 3 or more persons.

### Behavior mapping

#### Spatial distribution according to activities, gender, and age group

Fig 3 shows point distribution maps for the HDA (upper) and the LDA (lower) allowing the identification of spatial patterns of behavior. Fig 3A shows the spatial distribution of the activities grouped according to the purpose (free time, mobility, and work). This map shows that one of the HDA streets concentrates the highest number of people engaged in both mobility and free time activities. The main physical characteristic of this street is that one of its sidewalks is wider and has more grassy areas and vegetation than the average of the streets observed in this study. On the other hand, in both HDA and LDA there is a grouping in specific zones of people dedicated to labor activities, related to the construction of buildings that were carried out in both study areas. Fig 3B shows the spatial distribution according to gender, where men and women seem to be homogeneously distributed except for the higher concentration of men in the two specific street sections where the work activities were clustered. Fig 3C shows the spatial distribution according to age groups, where it is possible to observe that the general predominance of adults is only attenuated in some stretches of streets. In the case of HDA, a greater presence of children and older adults was observed along the sidewalk that stood out for having more areas with grass and vegetation. In LDA, this pattern change was only observed in one of its street sections (top left).

#### Point distribution, Kernel density, and analysis by street segment

Behavior mapping allows the identification of general patterns of behavior at different spatial scales, including the neighborhood area, streets, street segments, and behavior setting. Fig 4A shows the original spatial distribution of the observations represented in points for the HDA (upper) and the LDA (lower). Fig 4B shows the Kernel density estimate for both sites, highlighting in red color and orange color the areas of the streets with the highest concentration of people. These maps facilitate the identification of areas of greater and lesser activity. In this case, a relationship is observed between a higher pedestrian density with three characteristics of public spaces, their physical attributes, such as a larger area of vegetation, proximity to commercial areas and the construction of buildings. Fig 4C shows the result for each street segment, a scale frequently used in physical audits of streets [1, 3] and that would allow relating its results to the observed activity.

#### Behavior setting

**Fig 5:**
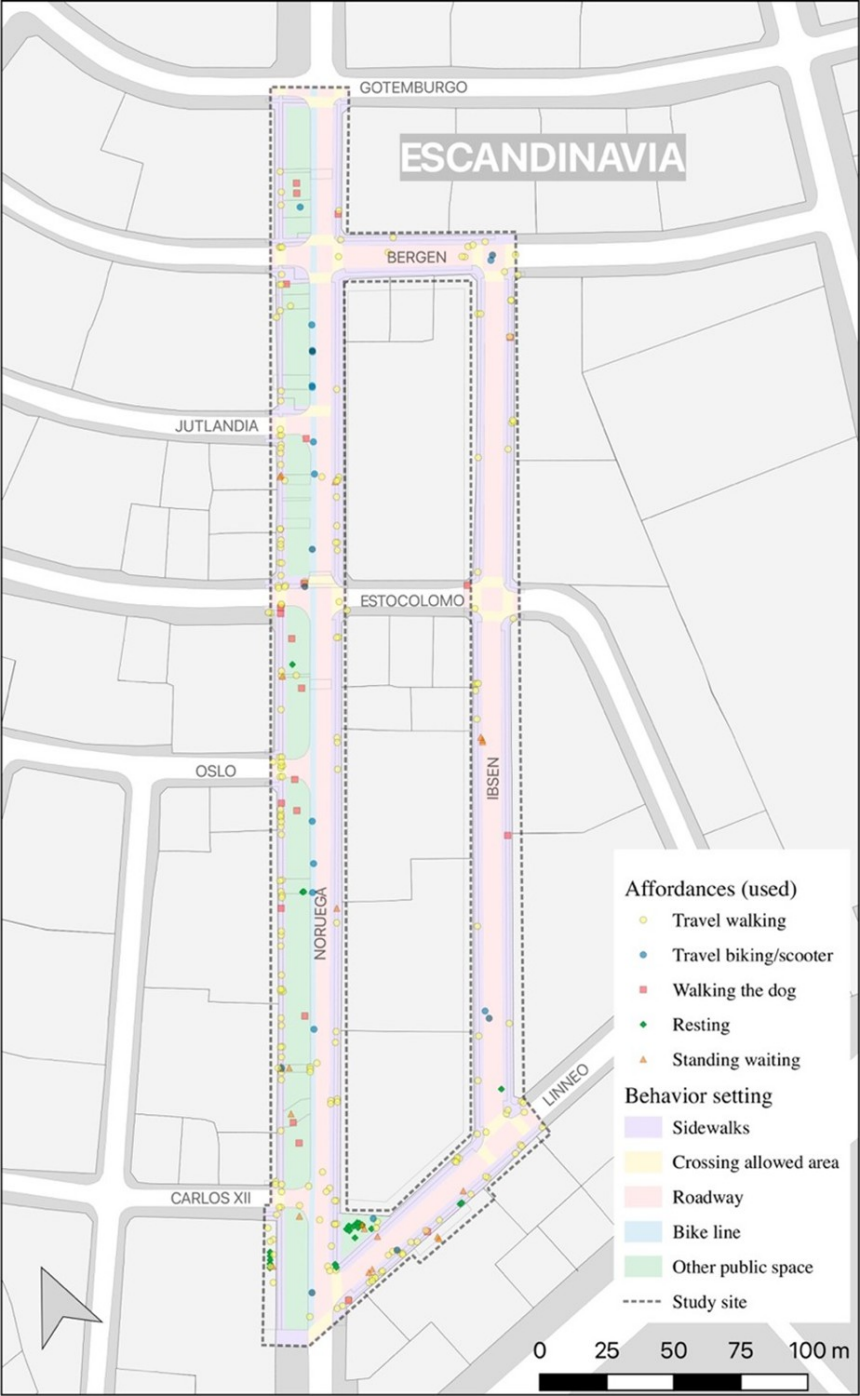
Behavior setting and active affordances. **Source**: own production over Openstreetmap data.

In Fig 5, it is possible to observe the spatial patterns of active affordances in the different behavior settings of the HDA. This image makes it possible to spatially identify possible conflicts between observed behavior and the street characteristics, such as the frequency of crossing through non-authorized areas of the street, a situation that could be occurring at the intersection between Noruega street and Carlos XII street.

**Fig 6:**
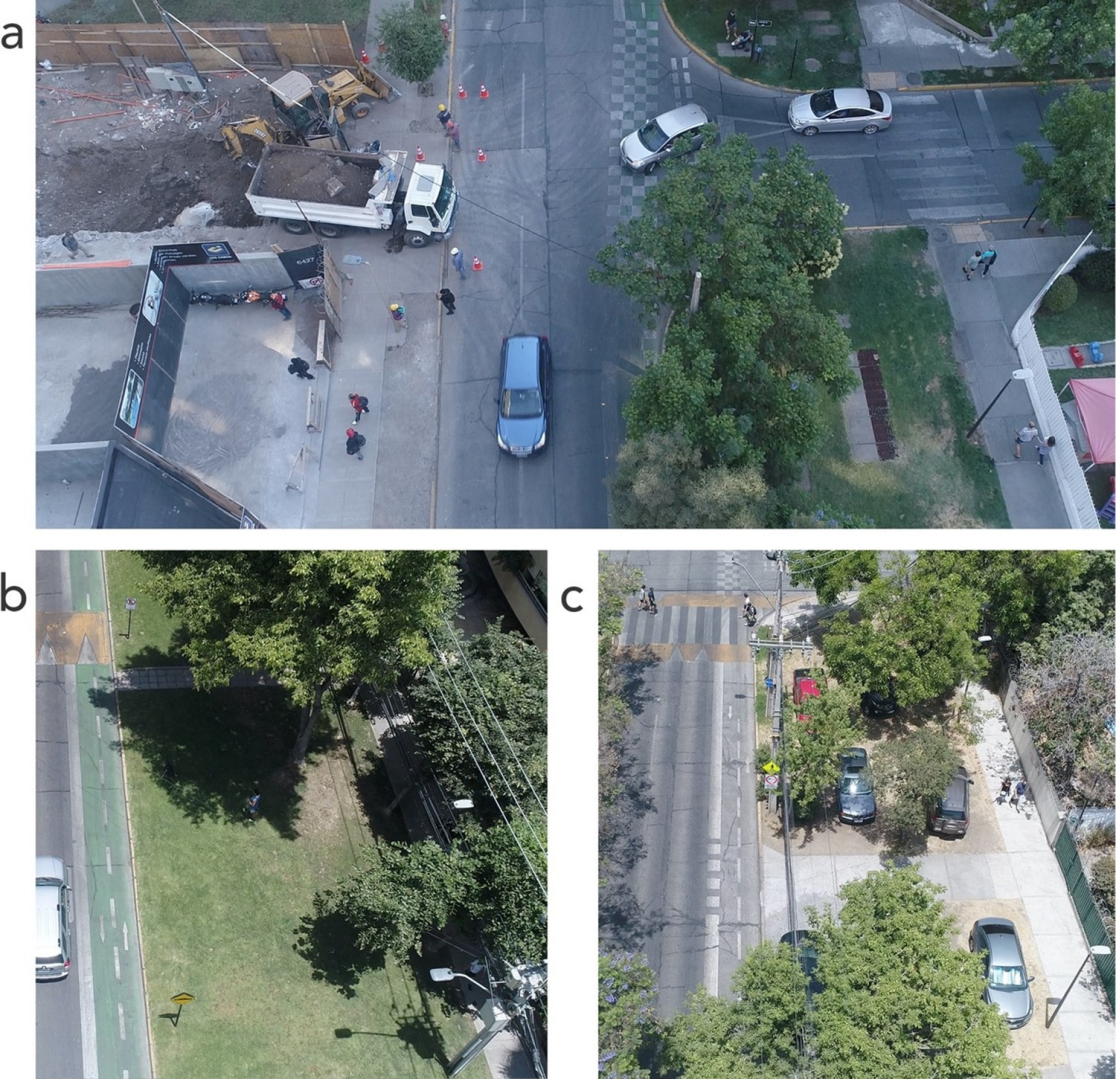
Intersection between Noruega and Carlos XII street (a). Grass and vegetation areas as improvised space for dogs (b). Grassy areas deteriorated by car parking (c). **Source:** original UAV imagery.

Table 3 shows the results of the active affordances in the different behavior settings of the HDA. On sidewalks, permitted crossing areas (trips on foot), and bike lanes (trips by bicycle/scooter) a dominant main activity was observed, however, in other public spaces and on roads, a greater diversity of activities was observed. For example, in other public spaces, such as grassy areas and vegetation between the sidewalk and the roadway, 38% of people rested sitting or lying down in them and 20% used them as places to walk their dogs (Fig 6). On the other hand, in addition to crossing the street walking (63%) and cycling (14.8%), 14.8% of people stood on them, usually waiting in the vicinity of a car.

**Table 3:** Behavior setting and active affordances.

Behavior Setting	(n) Active affordances	% travel walking	% travel bike/scooter	% walking the dog	% resting	% standing waiting
Sidewalk	184	87.0	0.0	5.4	2.2	5.4
Other public spaces*	50	24.0	2.0	20.0	38.0	16.0
Crossing allowed area	22	100.0	0.0	0.0	0.0	0.0
Bike line	13	7.7	92.3	0.0	0.0	0.0
Roadway	27	63.0	14.8	3.7	3.7	14.8
Total	296	71.6	5.7	7.1	8.1	7.4

* Area between sidewalk and roadway and other small green areas.

## Conclusion

This study proposes an alternative technique to observe the behavior of people on the streets, based on the advantages of an UAV in data acquisition and in the adaptation of behavior mapping techniques. This study has shown that the reliability between two raters who reviewed the same videos acquired by the UAV is high, and this has been verified in previous studies [40]. Our method benefits from the recognized advantages of UAVs over conventional data collection techniques, such as the elevated position of the observation point, the constant speed of UAV flight, and the backup of data with its subsequent revision that the videos acquired on the flight allow. Based on these attributes, the study shows that the use of UAVs allows us to expand the register of the volume of pedestrians with additional information that can improve our understanding of how streets are actually used, such as the specific activities of people and their interactions with objects, animals, or other people. These advantages are very useful in using behavior mapping to understand spatial patterns, or in the different geospatial analyses that the GIS allows, some of which have been exemplified in this study. In addition, our analysis shows that by using UAVs it is possible to simultaneously observe people on both sides of the street, and even the activity that occurs on the road which, together with the possibility of traveling at a speed higher than that demanded by other techniques, can result in significant resource savings. Finally, the proposed method allows observation based on momentary time sampling to be carried out with less time differences between the different parts that make up a study area, unlike other studies carried out on foot, where differences of several minutes or even hours often occur between the start and the end of the same observation route [53, 54].

However, there are some limitations that make it difficult to observe the behavior of people on the street using the UAV. The aerial observation of our methodology allows to overcome visual obstacles that occur at ground level, such as large parked vehicles, although tree foliage made it difficult to recognize people in some street sections. In addition, the available batteries for powering the UAV determine the maximum observation time. Weather conditions can restrict operations, such as rain, high winds, or night flights, but while the time required for observation in the study areas decreases, the subsequent coding time increases. Nevertheless, one of the most determining limitations is found in the operational rules defined by the local aviation administration, which for our case we have noted in the methodology section. Drone studies have been conducted on urban streets in different countries, such as the US, China, or Thailand [39, 40, 47], but there are countries or urban areas where it is not possible to use the UAV. Notwithstanding, these rules are prone to changes or updates, such as those recently made to the European regulations by the European Union Aviation Safety Agency (AESA) in 2021, whose new regulations would allow the use of UAVs in urban areas, requiring the authorization of AESA for operations where there are concentrations of people. In summary, to overcome the limitations when observing pedestrian activity using unmanned aerial vehicles, it is important to carefully select the right type of UAVs, to be compliant with regulations, to have a secure data management strategy, to plan for battery limitations, and to be aware of weather conditions. Additionally, it is important to be open to alternative methods and technologies, such as stationary cameras, ground-based sensors, or crowdsourcing, if the limitations of UAVs become prohibitive.

Together with the other direct observation techniques, the method that is the focus of this article has proven to be reliable in the evaluation of personal characteristics such as gender or age group, which are generally some of the variables that obtain the lowest results among evaluators, and in our case it was appreciation of the older adult category, hence we recommend further training to unify evaluation criteria.

One of the limitations of this study is the selected streets, which may not be representative of the reality of the streets in the cities of Chile, with Santiago de Chile being a city characterized by strong socio-spatial inequalities. Since the observed streets of Santiago de Chile correspond to an area of the city whose renovation was promoted by real estate businesses and which subsequently transformed the urban landscape thus presenting characteristics that are similar to those in modernized areas of many Latin American metropolises and the Latin American globalized world [55].

Although the main objective of this study was to analyze the proposed methodology and demonstrate its usefulness with a spatial analysis of the street behavior data, we recognize the importance of including the study of the temporal dimension. Future research would benefit by expanding the hours, days, and season selected for observation, if the objective were to characterize the temporal patterns of different activities in detail and the various street users. In this sense, given that the batteries only allow momentary observations, it is recommended to have several batteries available for observations at different times of the day.

Finally, this method has no substitute in terms of objectively understanding what people really do, hence it is noteworthy that the important improvement which UAVs provide to direct observation can help planners and decision-makers to better understand the patterns of use of space. An important issue where we believe that this methodology can be useful is in the validation of instruments that assess the quality of the built environment (such as audit tools, checklists, or urban indices), helping to identify which urban characteristics support or hinder the use of the street with respect to different user groups for different purposes.
